# An Open and Transferable Deep Learning Framework for Mapping Urban Tree Canopy Using NAIP Imagery

**DOI:** 10.3390/rs18121899

**Published:** 2026-06-09

**Authors:** Jooyoung Yoo, Yi Qi, Isaac Ashe-McNalley, Beau MacDonald, John P. Wilson

**Affiliations:** 1 Department of Computer Science, Emory University, Atlanta, GA 30322, USA; 2 Spatial Sciences Institute, University of Southern California, Los Angeles, CA 90089, USA

**Keywords:** urban trees, NAIP imagery, deep learning, reproducibility

## Abstract

The urban tree canopy is an important resource that spans public and private property and whose form and quantity change over short distances. Although remote sensing and deep learning approaches have been used to map urban tree canopy, the high cost of commercial imagery and the technical complexity of model development have limited their adoption by urban forestry practitioners. We developed a structured and reproducible deep learning workflow optimized for freely available USDA National Agriculture Imagery Program (NAIP) imagery. The workflow incorporates a reproducible U-Net segmentation model for canopy delineation and a YOLOv9e object detection model for individual tree identification, enabling complementary estimation of the canopy extent and individual tree locations. Across two neighborhoods in Los Angeles, the optimized U-Net achieved a Dice coefficient of 0.824 for canopy segmentation, while YOLOv9e reached an F1-score of 0.687 for individual tree detection on a held-out test set with 17,466 annotated trees. A data sufficiency experiment showed that model performance stabilizes when approximately 130 trees are annotated per 320 × 320 pixel (px) tile, corresponding to about 25,379 training and 2641 validation labels, providing a practical target for annotation effort. Additional experiments demonstrate a structured workflow for spatial sampling, training data requirements, and the use of model inferences to estimate tree canopy extent and individual tree locations. The workflow also shows encouraging evidence of transferability to previously unseen urban areas without retraining. By relying solely on NAIP-optimized approaches, this new workflow bridges the gap between complex deep learning techniques and the practical needs of urban foresters; empowers local stakeholders to create accurate, affordable, and timely urban tree inventories; and fosters data-driven decision-making for the sustainable management of urban green infrastructure.

## 1. Introduction

Urban trees represent a critical component of green infrastructure, delivering essential ecosystem services such as thermal regulation, infiltration, stormwater runoff reductions, air pollution mitigation, and the alleviation of heat-related health risks while contributing to decreased energy consumption [1–3]. Despite their well-documented benefits, urban trees are increasingly vulnerable to a range of biotic and abiotic stressors, including plant diseases; insect infestations, extreme meteorological events, shifts in temperature, precipitation and aridity levels; and wildfire susceptibility. These environmental stressors threaten urban tree health and the critical ecosystem services that trees provide [4,5]. McDonald et al. [6] documented the disparities in urban tree cover across 5723 U.S. municipalities and the correlations with summertime heat-related spikes in mortality, morbidity, and electricity consumption. Their work points to the need for systematic, recurring assessments of urban tree populations to support data-driven decisions in urban and community forestry planning and management.

Traditionally, municipal tree inventories have been compiled by professional arborists through field surveys, capturing a variety of tree attributes such as location, species, height, diameter at breast height, wood condition, and mortality status on streets and in parks and other types of public spaces [7,8]. While traditional inventories provide valuable and precise in situ measurements, their high cost and time-intensive nature hinder their feasibility for large-scale systematic monitoring and repeated assessments over time. The use of these methods to capture the trees on private property is also rare and would likely take much more time and effort in most cities around the world [9].

In response to these challenges, photogrammetry and remote sensing methods have been increasingly adopted and used to facilitate urban tree inventories because they offer streamlined workflows, improved data accuracy, enhanced update frequency, and reduced costs. The early studies utilized land-use and land-cover datasets, such as the National Land Cover Database (NLCD), derived from 30 m Landsat imagery, to evaluate urban tree cover changes at regional and national scales [10]. However, moderate-resolution imagery presents challenges in accurately detecting urban trees due to the heterogeneity and complexity of urban landscapes. High-resolution urban tree maps are crucial for practical forest management [11] and social research related to urban tree cover [12].

More recently, the focus has shifted towards the use of meter- and sub-meter-resolution imagery acquired from diverse spaceborne, airborne, and street-level sensors. High-resolution satellites, such as SPOT [13], Sentinel-2 [14], WorldView [15], PlanetScope [16], and Pleiades [17], have been extensively used for mapping urban tree characteristics. Additionally, aerial multispectral [18], lidar [19,20], and hyperspectral sensors [21,22] along with Google Street View imagery [23,24], and multi-sensor fusion approaches [25] have further enhanced urban tree mapping capabilities. The integration of these diverse remote sensing technologies offers increased spatial precision and analytical capabilities, and the opportunity to support more effective urban forestry management and policy development.

There has been a paradigm shift toward deep learning methodologies for analyzing and interpreting remote sensing imagery [26] in recent years as well. Image segmentation [27] and object detection [28] models have demonstrated superior capabilities in processing complex, heterogeneous, high-dimensional, and high-resolution remote sensing data. These advanced techniques facilitate more precise and detailed mapping of urban trees and green spaces [29]. A wide range of models based on CNN architectures have been employed to segment complex urban tree features, such as UNet, Mask R-CNN, self-supervised vision transformers, DeepLabv3+, and the Segment Anything Model [19,30–38].

Among these models, U-Net is a fully convolutional encoder/decoder structure designed for thematic segmentation, addressing the image localization challenge in CNNs by incorporating encoder pathways and decoder paths in a U-shaped architecture. Wagner et al. [33], for example, used a U-Net model for regression to map the canopy height of all trees across the state of California using canopy height models computed from aerial lidar data along with corresponding RGB-NIR NAIP images. Li et al. [39], on the other hand, developed a U-Net framework integrating high-resolution aerial imagery and lidar data to detect individual overstory tree locations, crown areas, and heights in Denmark.

Turning next to object detection, YOLO has emerged as a widely utilized single-shot object detector known for its efficiency and strong performance in real-time applications. Choi et al. [40], for example, leveraged YOLO for tree species detection and profile estimation of urban street trees using Google Street View imagery. Since its inception in 2016, multiple updated versions have been developed to enhance detection accuracy and training stability. Recent versions after 2024, particularly YOLOv9e, introduce architectural and training advances such as Programmable Gradient Information (PGI) and a GELAN backbone, which improve feature representation and stabilize training. These enhancements are especially beneficial for detecting small, densely distributed objects with variable shapes—characteristics typical of individual tree crowns in remote sensing imagery. While later versions (e.g., YOLOv10 and YOLOv11) emphasize incremental engineering refinements, YOLOv9e provides a strong balance between detection accuracy, computational efficiency, and robustness. These properties make it a practical and well-suited choice for individual tree detection in high-resolution aerial imagery [41,42].

Notwithstanding the advances made using remotely sensed imagery and deep learning methods to map urban trees, there are at least three limitations that hinder their widespread adoption and use among practitioners, such as city foresters and arborists, who require scalable and efficient solutions for real-world management and assessments across diverse urban environments [43].

The first limitation of using deep learning models to map urban trees focuses on reproducibility, generalizability, and repeatability [44,45]. Deep learning model development typically requires extensive expertise in model selection, programming, computational infrastructures and hyperparameter optimization, which can present significant technical challenges for non-experts [46]. Despite rapid advances in deep learning for remote sensing, many proposed workflows remain difficult to reproduce in practice because code, data preprocessing steps, and hyperparameters are not fully documented or openly shared. This reproducibility gap is particularly problematic for municipal forestry agencies and other practitioners who lack the resources to build and/or use complex GeoAI pipelines from scratch. In addition, many of these models are trained using data from specific geographic areas, raising concerns about their effectiveness and applicability to different locations and a broader range of urban tree characteristics. The tree detection models recently developed for California [38], for example, may not reflect the variability in different urban landscapes, making them less reliable for local decision-making. To better serve local urban forestry initiatives, a “bottom-up” approach is needed, in which practitioners develop repeatable workflows tailored to their specific environments. Such an approach would allow the creation of local deep learning models that can map urban trees at preferred temporal intervals while maintaining reliable accuracy and manageable computational costs. By fostering the development of adaptive and community-driven methodologies, urban tree mapping can become more accessible, actionable, and scalable for a wide range of stakeholders.

The second limitation of developing deep learning models for mapping urban trees is the choice and cost of remotely sensed imagery. Although a wide range of high-resolution remote sensing imagery exists to support urban tree mapping, much of this imagery is gathered by commercial satellites and aerial sensors, both of which require substantial financial resources and specialized expertise for data acquisition, management, and processing. The primary need for urban forest managers and practitioners is the availability of affordable, seamless, and recurrent data collection methods for large-scale tree inventory development. NAIP has emerged as a promising and free aerial multispectral remote sensing dataset for urban tree mapping, offering nationwide coverage, a recurrent acquisition cycle (every 2–3 years), high spatial resolution (60 cm to 1 m), multispectral imaging capabilities (RGB and NIR bands), and a historical archive dating back to 2003 [18,47]. This imagery has several inherent challenges, including variations in illumination conditions and sensor viewing angles, inconsistencies in image georegistration, phenological differences in vegetation, and spectral mixing from background and adjacent objects [48]. It is therefore imperative to develop tailored workflows that mitigate these challenges to fully encapsulate the value of NAIP imagery for widespread adoption.

The third limitation is the substantial volume of training data required to train and validate deep learning models, which often depend on time-intensive field measurements and manual annotation. Such datasets are typically unavailable or unaffordable in many local communities, particularly in low-income and underrepresented urban areas. Even when data collection is feasible, practitioners face challenges in selecting appropriate sampling methods and determining the optimal dataset size within budget and time constraints. Potential biases stemming from data imbalance or annotation errors may significantly impact model validity [49], which speaks to the importance of choosing spatially random sampling strategies for building training data for urban forest canopy mapping applications [50,51]. In addition, an insufficient amount of training data can lead to model overfitting and reduce its ability to generalize and transfer to new tasks. Recently, data augmentation techniques have been explored to address the scarcity of annotated data in Earth observation and to improve the performance of remote sensing-based models [52]; however, practical guidance on training data collection methodologies remains essential to support localized efforts in model development and implementation.

Taken together, these limitations highlight the need for a practitioner-oriented urban tree mapping workflow that is not only methodologically robust but also operationally accessible and scalable. To reduce the financial costs associated with remote sensing data acquisition across the United States, we deliberately propose the adoption of freely available NAIP imagery as the primary data source, rather than relying on costly high-resolution lidar or commercial aerial imagery. NAIP provides an effective balance of spatial, spectral, and temporal resolution, along with nationwide coverage and a long-term archive, making it well suited for structured, repeatable and scalable urban tree canopy mapping. Moreover, fully realizing the potential of NAIP imagery requires workflows that are explicitly optimized for NAIP data and capable of addressing the technical complexities associated with deep learning model development. Although prior studies have applied a range of deep learning models to NAIP and similar imagery, practitioners’ adoption is driven less by model novelty than by the clarity and reproducibility of the workflow, which enable consistent implementation and predictable performance. In particular, practitioners lack NAIP-specific guidance spanning training data sampling and preparation, model configuration and hyperparameter tuning, and the outcomes provided by operational urban tree inventory workflows. This study addresses this gap by developing a NAIP-optimized, reproducible, and standardized deep learning workflow that lowers technical and financial barriers for practitioners while enabling consistent and transferable application across diverse urban environments. Broader adoption of such deep learning workflows has the potential to support more systematic urban tree monitoring and to facilitate extensions in transfer learning methodologies within remote sensing [53] and expand applications from single- to multi-city analyses [54].

Building on this rationale, the overall goal of this study is to develop a NAIP-optimized, reproducible, and standardized deep learning workflow for urban tree canopy mapping that can be readily adopted by practitioners in American cities. Rather than proposing a new model architecture, we focus on integrating complementary methods into a coherent, end-to-end workflow that supports two core tasks in urban forestry: delineating canopy extent and identifying individual trees. Specifically, we (i) incorporate a U-Net segmentation model and a YOLOv9e object detection model within a single reproducible workflow, in which the models are trained and inferred independently to estimate canopy area and detect trees; (ii) systematically evaluate the effects of key design parameters, including tile size, threshold selection, data augmentation, and training data sufficiency, to establish empirically grounded best practices for NAIP-based modeling; and (iii) assess the potential transferability of the workflow across previously unseen urban environments. Through this approach, the study aims to provide both a validated workflow and actionable guidance for scalable and consistent urban tree canopy mapping.

## 2. Materials and Methods

**Figure 1.**
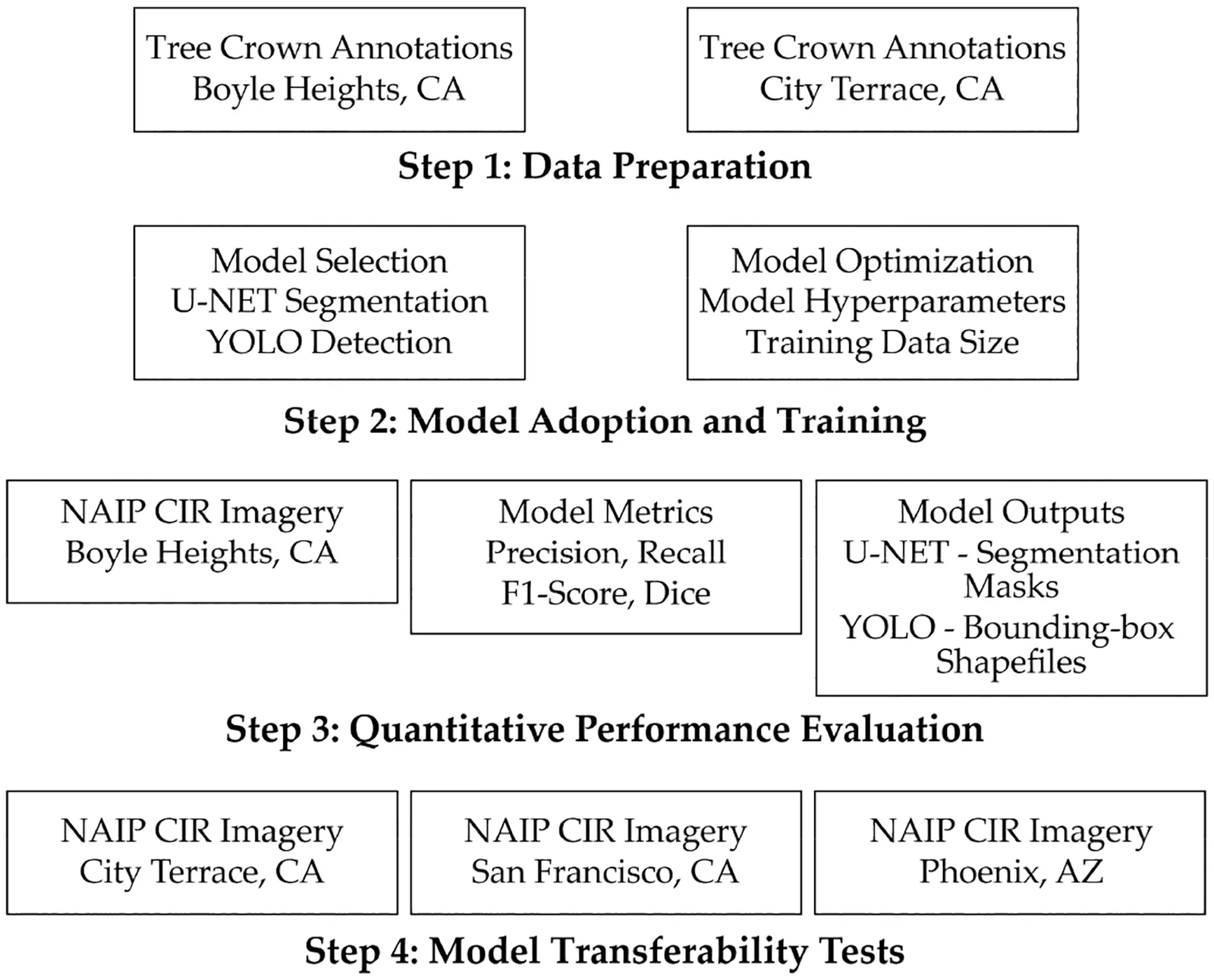
The study workflow.

We adopted a structured research design to systematically develop, evaluate, and demonstrate a reproducible, open, and transferable deep learning framework for mapping urban tree canopy using publicly available NAIP imagery. The workflow, illustrated in Figure 1, was organized into four main stages: (1) data preparation; (2) model adoption and training; (3) quantitative performance evaluation; and (4) training data size and model transferability analysis. Each stage was designed to address key challenges and ensure the final framework was both scientifically robust and capable of advancing the work of urban forestry practitioners.

### 2.1. Data Preparation

**Figure 2.**
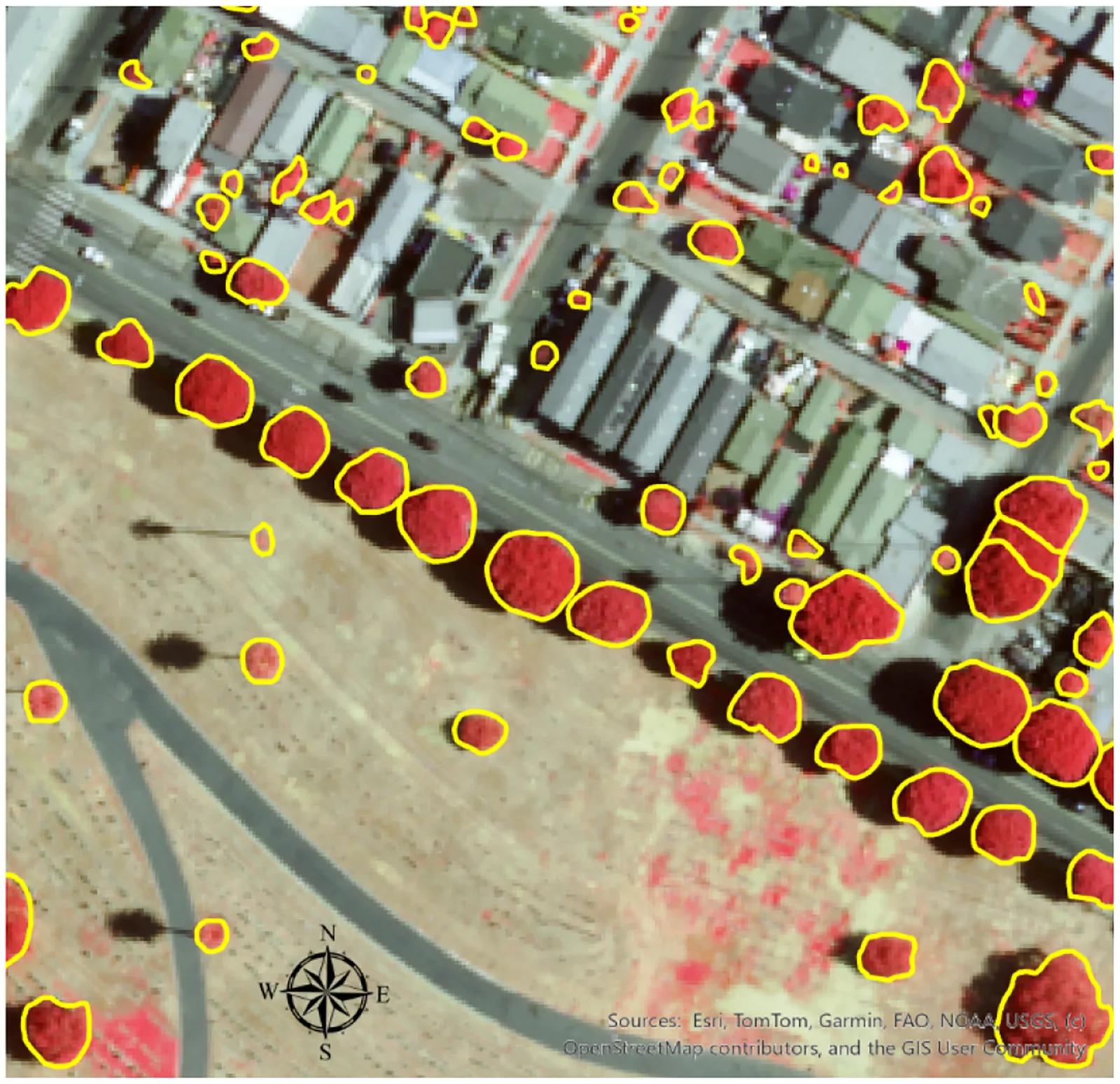
An example of manual tree crown annotations on a NAIP CIR image in a 320 × 320 px tile. Yellow outlines mark manually labeled tree crowns that formed both the training and test datasets.

The first stage focused on preparing USDA NAIP color-infrared (CIR) imagery and corresponding manual annotations for the deep learning models (Figure 2). A spatially stratified random sampling strategy was implemented to ensure a rigorous and unbiased evaluation of the model’s ability to delineate the tree canopy. All the annotated data for Boyle Heights was allocated for model training and validation, using a random 90/10 percent split. We used the City Terrace data to evaluate the model’s performance in an unseen geographic area. To improve model robustness and prevent overfitting, a series of data augmentation techniques was applied to the training dataset. These transformations included random horizontal and vertical flips, 90° rotations, and adjustments to image brightness, contrast, and saturation. By artificially expanding the diversity of the training data, augmentation also improved the capabilities of the models to handle the natural variations in lighting, seasonal conditions, and sensor angles in the NAIP imagery acquired for different regions and years.

In total, 28,020 tree crowns were manually annotated in the Boyle Heights study area. Of these, 25,379 annotations (90%) were used for model training and 2641 annotations (10%) for validation, following a random tile-based split. All tree crown annotations were performed by trained research staff with backgrounds in urban forestry and GIS, following a structured protocol that defined crown boundaries as the visible outer canopy edge in CIR NAIP imagery [55]. Ambiguous boundaries due to occlusion or crown overlap were delineated based on the most visually identifiable edge. Point-based tree location data provided by Los Angeles County were overlaid and cross-referenced with Google Maps satellite imagery to further enhance annotation accuracy. Quality control involved independent review by a second team member, with discrepancies resolved through discussion. For external evaluation, an independent City Terrace dataset was used, consisting of 17,466 manually annotated tree crowns distributed across 100 image tiles. This dataset was held out entirely from model development and hyperparameter tuning and used exclusively for testing.

**Figure 3.**
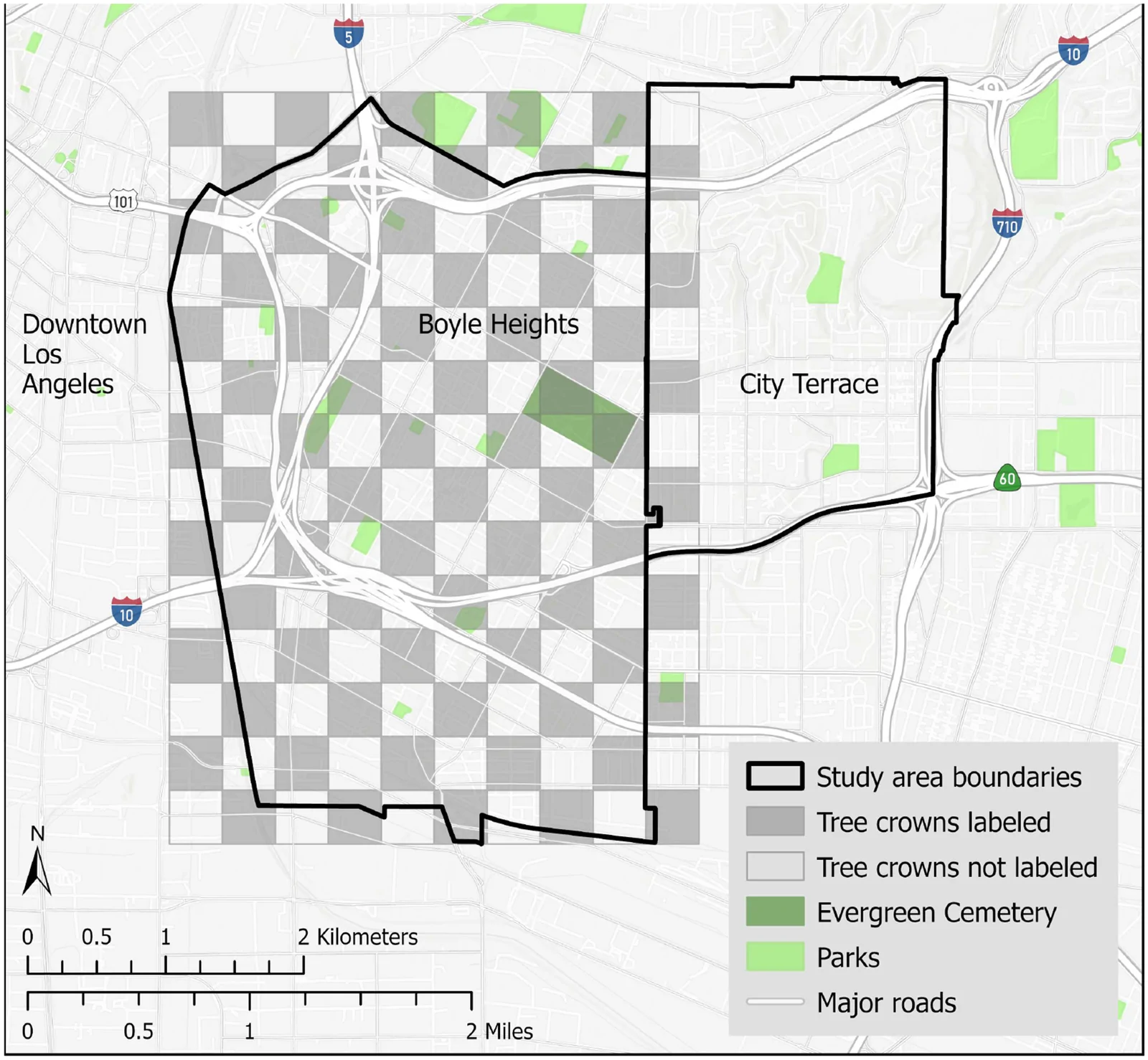
A map showing the Boyle Heights (**left**) and City Terrace (**right**) study areas with major roadways, parks and the Evergreen Cemetery. The white-shaded tiles in Boyle Heights show the areas in which tree crowns were labeled manually.

The two study sites—Boyle Heights and City Terrace—are located east of Downtown Los Angeles (Figure 3). Boyle Heights is a poor, mostly Latino residential neighborhood with a storied history that supports single- and multi-family housing along with commercial and light industry along its southern and western borders. City Terrace is an unincorporated area in Los Angeles County located immediately east of Boyle Heights. This is also a poor, mostly Latino residential neighborhood but it has more hills than Boyle Heights and many of the streets lack sidewalks and therefore street trees. Kianmehr et al. [56] used individual tree growth and longevity data to portray the current and future conditions of street trees in these two and four adjacent neighborhoods and found that nearly half of the current tree canopy will disappear by 2050, and on average, approximately one percent of the existing street trees will be lost due to aging each year.

### 2.2. Deep Learning Architectures

This study sought to develop models for two common metrics found in urban forest inventories: tree presence and canopy coverage. While a wide range of image segmentation models (i.e., Mask R-CNN, YOLO-Seg, and zero-shot models), dual-model frameworks [57,58], and recently an image scene classification model [59], have been used to map individual trees, these approaches are usually paired with very-high resolution imagery at centimeter level or lidar data, which are not widely available in many parts of the world. The success of urban tree delineation depends not only on image characteristics and model strategy, but also on crown size, spectral contrast, and urban landscape structure. The spatial resolution of NAIP imagery (60 cm to 1 m) presents challenges for delineating individual tree crowns, particularly in urban contexts where crowns are small, spectrally similar to surrounding surfaces, or densely clustered. Our preliminary experiments using Mask R-CNN (ResNet-152 backbone; Precision = 0.347) and YOLOv9-Seg (Precision = 0.704, Recall = 0.564) resulted in inferior performance relative to the U-Net and YOLOv9e models and were therefore not pursued further in this study. As such, NAIP data is better suited for identifying tree canopy clusters and supporting tree detection tasks using models like U-Net or YOLO.

We selected the U-Net architecture, a fully convolutional neural network renowned for its efficacy in biomedical and satellite image segmentation, to delineate tree canopy cover. U-Net with an encoder based on ResNet50 has been widely and successfully applied to high-resolution land-cover and vegetation segmentation tasks in remote sensing, where its skip connections help preserve fine spatial detail while capturing broader contextual information. Building on these studies, we adopt a ResNet50 backbone pre-trained on ImageNet as the encoder to leverage robust multi-scale features and to reduce the amount of task-specific training data required. This allows the model to harness robust, hierarchical features learned from a massive dataset to improve its feature extraction capabilities. Skip connections between the encoder and decoder paths ensure that high-resolution feature details from early layers are propagated to deeper layers, enabling the model to generate fine-grained segmentation masks that accurately outline tree canopies. U-Net and related encoder–decoder models have been successfully applied to high-resolution aerial imagery and NAIP-based tree mapping, including lidar-informed U-Net models for sub-meter canopy height mapping across large regions. These studies indicate that U-Net is a strong baseline for representing complex urban tree structures in NAIP imagery [60].

We used the YOLOv9e model, a single-stage object detector, to identify and locate individual trees. Recent evaluations of YOLO variants on aerial and remote sensing imagery have shown that these architectures perform particularly well for detecting small objects under varying viewing geometries and backgrounds, due to their enhanced feature aggregation and multi-scale prediction heads. YOLOv9e, in particular, incorporates a programmable gradient information mechanism and a GELAN-style backbone that improves convergence and small-object sensitivity, making it a suitable choice for detecting tree crowns that often occupy only a small fraction of NAIP tiles [61]. YOLOv9e processes entire images in a single pass, making it exceptionally fast and efficient. It predicts both bounding boxes and class probabilities simultaneously, framing object detection as a regression problem. Its architecture incorporates advancements such as a C2f (Cross-Stage Partial-fusion) backbone and a decoupled head, which improves accuracy compared to previous versions. Furthermore, for this study, YOLOv9e was configured to detect a single class, “tree,” outputting bounding box coordinates for each detected tree canopy within an image tile. We also evaluated several alternative architectures, including a segmentation variant of U-Net++, SegFormer, and DeepLabV3, but found that the U-Net had the most robust performance in our experiments (see Section 3.2).

The U-Net and YOLOv9e models were trained on a workstation equipped with an NVIDIA RTX 4000 Ada GPU using the PyTorch 2.10 deep learning framework. The YOLOv9e model was implemented through the PyTorch-based Ultralytics framework (https://www.ultralytics.com/). The learning rate for both models was set to 0.0001, and a batch size of 4 was used throughout the training. The U-Net model was trained for 50 epochs, while the YOLOv9e model was trained for 200 epochs. During training, model performance on the validation set was monitored to identify and save the checkpoint with the highest F1 score or Dice coefficient. The F1 score is often used to assess performance by combining precision and recall in a single metric to evaluate how well the object detection model (YOLO) balances false positives and negatives. The Dice coefficient serves a similar purpose for the segmentation tasks (U-Net), by quantifying the overlap between predicted and ground truth masks, providing a clear measure of how accurately the model captures the target regions. The Adam optimizer was selected for both models to leverage its adaptive learning rate capability, and a composite loss function combining Binary Cross-Entropy and Dice Loss to enhance segmentation performance (U-NET) [62]. The YOLOv9e model relied on its built-in loss function, which includes classification, regression, and distribution focal loss components. The U-Net architecture was kept as originally defined, and a sigmoid activation function was applied to the output logit map at the inference stage to convert the results into pixel-wise probability values ranging from 0 to 1.

We designed a suite of experiments to identify the important hyperparameters in U-Net and YOLO for optimal model training. The effect of input tile size on segmentation performance was first evaluated to establish a foundational model architecture. Four tile sizes (i.e., 80 × 80, 160 × 160, 320 × 320, and 640 × 640 pixels) were assessed under identical training conditions, without data augmentation or inference overlap, to isolate the impact of spatial context. The Dice score served as the primary evaluation metric for the semantic segmentation models, and the F1-score served as the equivalent metric for the object detection tasks. Following the selection of tile size, the next experiment aimed to determine the optimal pixel-probability threshold (τ) for post-processing in the U-Net model and the IoU and confidence thresholds in the YOLO model. While the baseline U-Net architecture was maintained, an external sigmoid function was applied to the model’s final output logit map to generate pixel probabilities from 0 to 1 for inference. This probability map was then binarized using a threshold (τ) to calculate performance metrics such as true positives (TPs), false positives (FPs), and false negatives (FNs). The threshold τ was explored experimentally across a range from 0.05 to 0.90. We then systematically evaluated the effects of applying a 50% overlap (160-pixel stride) and a comprehensive data augmentation framework. The overlap training helped the model learn more consistently near tile boundaries, which might otherwise lead to artifacts when the image is split into tiles.

One of the practitioners’ barriers to train deep learning models for generalizable application is the limited training data size. To improve model robustness and prevent overfitting, the training data underwent a sequence of augmentations, including horizontal flips and random 90° rotations (*p* = 0.5), to introduce geometric variation. ColorJitter (*p* = 0.5) was used to adjust brightness (±20%), contrast (±20%), saturation (±20%), and hue (±5%). CLAHE (*p* = 0.3) was applied with a clip limit of 4.0 and 8 × 8 px tiles to enhance local contrast. RGBShift (*p* = 0.3) introduced small random shifts in color channels (R: ±15; G: ±5; B: ±5). By artificially expanding the diversity of the training data, augmentation also helps the models to better handle natural variations in lighting, seasonal conditions, and sensor angles found across NAIP imagery acquired from different regions and years. Finally, all images were normalized to a zero mean and unit variance, and four configurations were tested: no overlap and no augmentation, overlap only, augmentation only, and both techniques combined.

The combination of the Adam optimizer with a Binary Cross-Entropy plus Dice loss has become a common choice for the semantic segmentation of high-resolution remote sensing imagery as it stabilizes training and directly optimizes overlap-based metrics such as Dice and IoU. Our augmentation strategy (random flips, rotations, color jittering, local contrast enhancement, and channel shifts) follows established practice in Earth observation, where such transforms help models generalize across heterogeneous illumination conditions, sensor viewing geometries, and seasonal variation. These experiments were conducted as a structured grid search over a predefined set of candidate values, tile size (80, 160, 320, 640 px), pixel-probability threshold τ (0.05 to 0.90 in increments of 0.05), overlap (0 vs. 50%), and augmentation (on/off), with each configuration evaluated on the held-out validation set. The results of these experiments were used to identify the NAIP-optimized steps in training data preparation and model training.

### 2.3. Quantitative Performance Evaluation

We conducted a comprehensive quantitative evaluation using task-appropriate metrics to assess model performance. The final models were built with the optimal hyperparameters and training data size noted earlier. For the U-Net segmentation model, evaluation was performed on both the Boyle Heights test dataset and the held-out City Terrace test set. Key evaluation metrics included precision, recall, and Dice calculated on a pixel-level basis. These metrics captured both the accuracy and completeness of the predicted tree canopy masks relative to the manually annotated ground truth. For the YOLOv9e object detection model, evaluation focused on bounding box accuracy using the City Terrace test dataset. We calculated and reported precision, recall, and F1 scores at the detection level. A prediction was considered a TP if the bounding box predicted by the model overlapped with a manually annotated tree crown based on a predefined Intersection over Union (IoU) threshold. FPs and FNs were also counted to evaluate the models’ over- and under-detection. These metrics provided a direct performance comparison between the two deep learning approaches (semantic segmentation and object detection) and informed tradeoffs in accuracy, granularity, and computational efficiency for practical deployment.

Given these approaches, it is important to note that Dice and F1-scores, while mathematically equivalent to the harmonic mean of precision and recall, operate at fundamentally different levels of analysis. Dice is computed from pixel-level TP, FP, and FN counts for the segmentation task, whereas F1-scores are derived from object-level bounding box matching for detection, using a predefined IoU overlap threshold. Direct cross-task comparisons between these metrics are therefore illustrative in nature rather than quantitatively equivalent and should be interpreted with this distinction in mind.

### 2.4. Training Data Size Analysis and Model Transferability

In addition to model training and evaluation, the study incorporates two practical tests that influence real-world applications.

We first examined how much training data is required to achieve reliable model performance. We conducted a data sufficiency experiment using the Boyle Heights test dataset to evaluate the impact of training dataset size on model performance. We used the number of trees per tile as a proxy for training data volume. Twelve configurations were tested with tree annotations ranging from 1 to 279 trees per tile in increments of 30. The performance metrics, which included precision, recall and Dice, were computed for each configuration. For each subset, the same data augmentation, model architecture, and hyperparameters were applied, and model performance was evaluated on the same validation and test datasets. To statistically test the impacts of training data size, we constructed 30 independent models for each of the 12 configurations and performed a one-way Analysis of Variance (ANOVA) on the F1-scores. This analysis revealed how model accuracy scaled with training data volume and helped identify the minimum volume of labeled data required to reach stable performance. The results provide practical guidance for practitioners aiming to implement deep learning models for tree canopy mapping with budget or time constraints.

The second test was executed—once the model training had been finalized with appropriate training data size and architecture—by applying the trained models on unseen NAIP imagery for Phoenix, AZ and San Francisco, CA. The former has arid landscapes with sparse tree cover, and the latter has a more temperate climate and varied tree canopy than our study area. The test sought to determine whether the models can produce accurate predictions in these settings without additional training. The models were applied without further fine-tuning, and their predictions were qualitatively assessed against visually interpreted reference images to learn how well the models could generalize canopy segmentation and tree detection patterns beyond the region they were trained in. We also compared the predictions with available municipal tree inventory data and vegetation maps to further assess external validity.

## 3. Results

The results below summarize the optimization of the model hyperparameters and the impact of training data size on model performance.

### 3.1. Model Hyperparameter Optimization

The effect of input tile size for U-Net and YOLO is summarized in Table 1. For U-Net, the 80 × 80 and 320 × 320 px tile sizes achieved the highest Dice score of 0.824, with comparable precision (0.790 and 0.789) and recall (0.862 for both). The 160 × 160 px configuration followed closely with a Dice of 0.823, while the 640 × 640 px tiles showed slightly lower recall (0.820) and Dice (0.815), despite having the highest precision (0.810).

When the U-Net outputs were used to guide the YOLO-based detection, the 320 × 320 px configuration yielded the highest F1-score (0.687). In contrast, the 80 × 80 px tile size, despite strong segmentation metrics, resulted in lower detection performance (F1-score: 0.681). Accordingly, we chose the 320 × 320 px tile size as the optimal configuration for the subsequent experiments to balance high segmentation accuracy and practical detection applicability.

The pixel-based probability threshold (τ) analysis revealed a clear performance plateau for the Dice coefficient at thresholds between 0.20 and 0.30 (Table 2). The highest Dice value (0.824) was achieved at τ = 0.25, with a precision of 0.789 and recall of 0.862. This threshold represents the optimal equilibrium between precision and recall. Lower thresholds (τ < 0.25) substantially increased recall but at the cost of reduced precision due to a higher rate of false positives; conversely, higher thresholds (τ ≥ 0.50) improved precision but markedly decreased recall, leading to significant omission errors. We therefore adopted and used the pixel-probability threshold of 0.25 for the remainder of the study.

We also evaluated the YOLOv9e model under four confidence and IoU threshold post-processing configurations. The evaluation was performed on the same City Terrace test dataset, which includes 100 image tiles and 17,466 manually annotated trees. The highest F1-score (0.687) was observed with an IoU threshold of 0.2 and a confidence threshold of 0.1, yielding a precision of 0.747 and recall of 0.636 (Table 3). The preferred model achieved a mean Average Precision (mAP@50) of 0.718, indicating reliable detection of individual trees under a moderate overlap threshold. The lower mAP@50–95 value of 0.436 reflected reduced performance under stricter localization criteria, highlighting the difficulty of accurately delineating tree crowns in complex urban environments and more generally, the practical challenges of remote sensing-based object detection [63]. Similar performance was found at the 0.2/0.2 threshold settings, with an F1-score of 0.685. The configuration with the lowest thresholds (IoU = 0.1, confidence = 0.1) achieved a balanced precision (0.679) and recall (0.686), resulting in a moderate F1-score of 0.681. The U-Net model demonstrated higher precision at the pixel level, whereas the YOLO model showed strength in object-level recognition, providing both the number of individual trees and the width of the largest tree and its precise location.

The results of applying overlap and data augmentation showed an interaction between the two techniques cast in four configurations: no overlap and no augmentation, overlap alone, augmentation alone, and both techniques combined (Table 4). The combined strategy of 50% overlap and data augmentation produced a more balanced precision–recall profile compared to augmentation alone, which substantially increased recall (0.907) at the cost of precision (0.719) and reduced Dice (0.802). The differences in Dice scores across the four configurations were relatively small. Applying overlap alone led to a minor precision gain (0.795) at the cost of a slightly reduced recall (0.850), resulting in a marginal drop in Dice from 0.824 to 0.821. In contrast, applying augmentation alone increased recall significantly to 0.907 but also caused a sharp drop in precision to 0.719 due to a rise in false positives, reducing the Dice score to 0.802. When both overlap and augmentation were applied together, the model achieved a more balanced performance with a precision of 0.801, recall of 0.845, and a Dice coefficient of 0.823.

**Figure 4.**
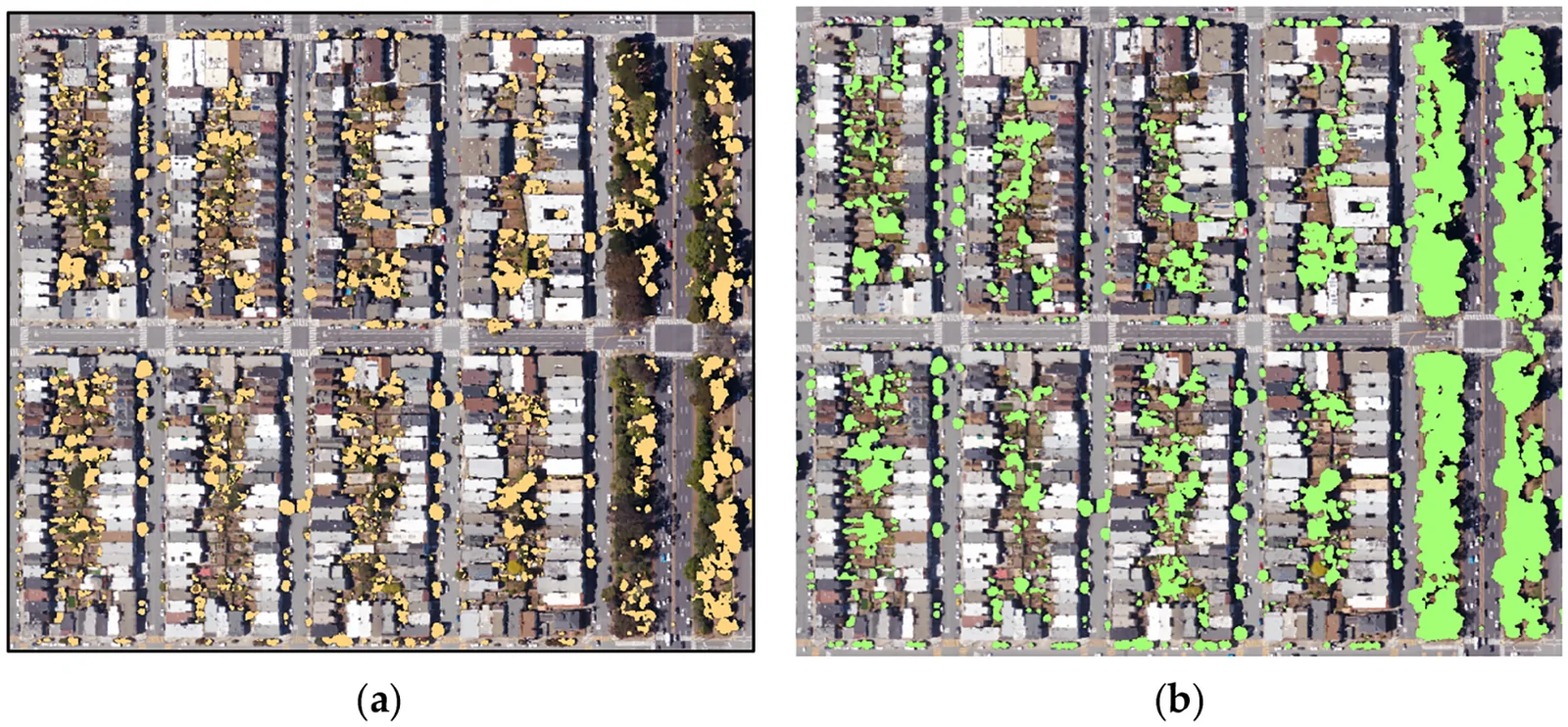
(**a**) This San Francisco, CA model, trained without data augmentation and overlap, failed to segment the dense urban tree areas, particularly along continuous rows of street trees, leading to substantial under-segmentation (orange overlay); (**b**) the model trained with data augmentation and 50% overlap produced more complete and accurate segmentation in high-density canopy areas, with improved coverage and boundary continuity (green overlay).

Although these numerical differences appear subtle, we conducted an additional qualitative evaluation on imagery for San Francisco, CA—an area far from the training and test regions—to assess model generalization. As shown in Figure 4, the differences in segmentation quality became significantly more visible in densely vegetated urban blocks. The baseline model without augmentation and 50% overlap failed to detect large portions of the tree canopy, particularly in areas with continuous rows of street trees and dense canopy clusters, resulting in widespread under-segmentation and FNs (Figure 4a). In contrast, the model trained with both augmentation and overlap produced more complete and coherent segmentation results, with improved boundary accuracy and coverage in high-density canopy zones (Figure 4b). These findings confirm that the combined strategy not only improved quantitative evaluation scores but also played a critical role in enhancing the model’s generalization to geographically unseen and structurally complex urban environments.

### 3.2. Model Comparison

To assess how our chosen architectures compare with other mainstream segmentation models, we trained U-Net, U-Net++, SegFormer-B0, SAM2, and DeepLabV3-ResNet50 under a consistent NAIP-based setup. U-Net achieved the highest Dice/F1 score (0.824), followed by U-Net++ (0.820, similar but slightly less stable across tiles), SegFormer-B0 (0.812), and DeepLabV3-ResNet50 (0.773). SAM2, however, tended to over-segment grass and other homogeneous green areas, generating many spurious detections; moreover, despite extensive fine-tuning, additional issues repeatedly appeared when transferring the model to other cities, so we ultimately excluded SAM2 from our framework. In addition, a YOLOv9-Seg model configured for canopy segmentation obtained a detection F1-score of 0.626 at an IoU threshold of 0.5 (precision 0.704, recall 0.564), and a Mask R-CNN model obtained precision = 0.347 (ResNet-152 backbone) which was lower than the performance of our U-Net and YOLOv9e models. Taken together, these results confirm that U-Net and YOLOv9e remain the competitive and robust choice for NAIP-based canopy segmentation and object (i.e., tree) detection in our setting.

### 3.3. Impact of Training Data Size on Model Performance

**Figure 5.**
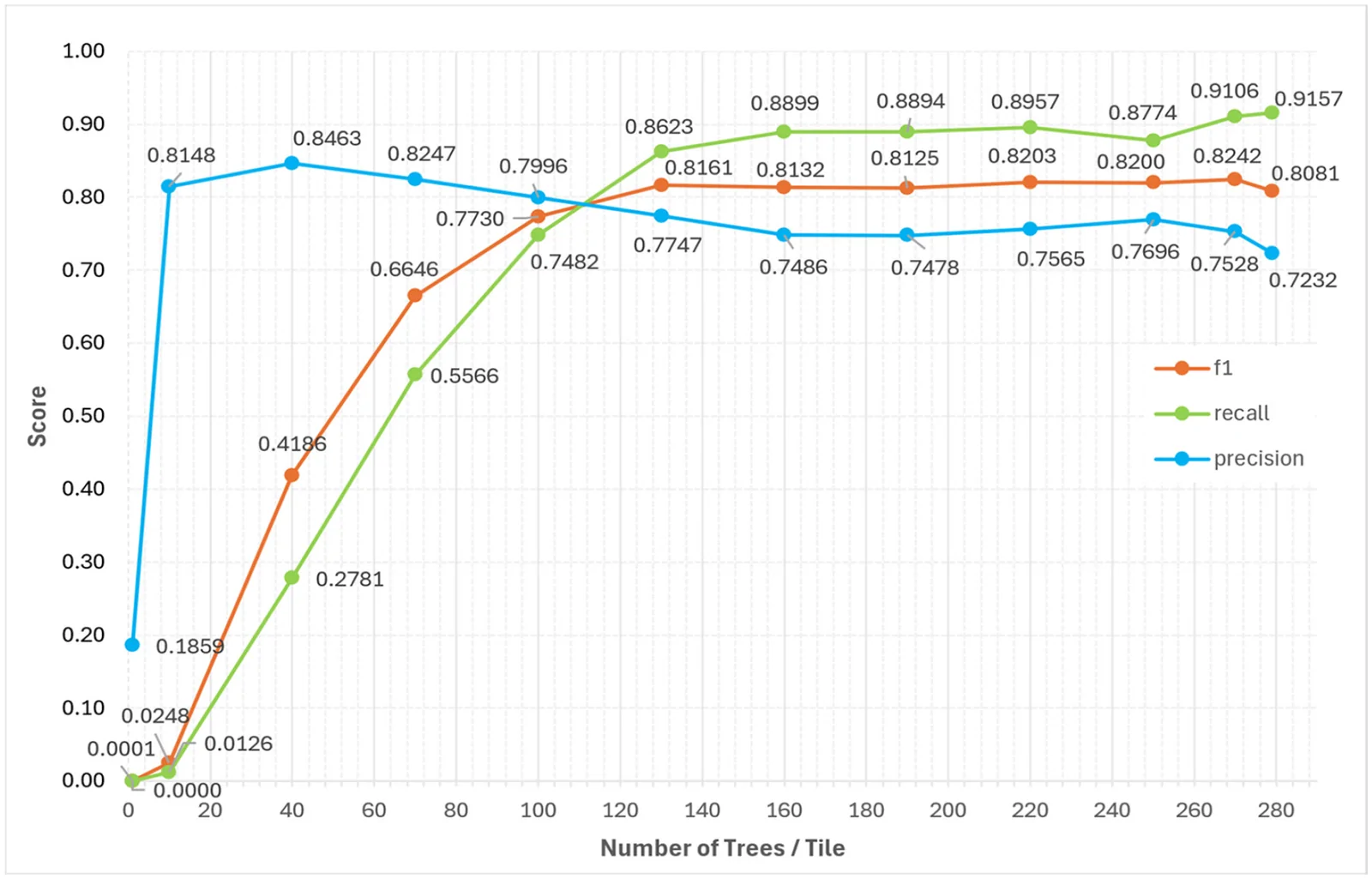
The variation in averaged F1, recall and precision for 30 models with increasing numbers of trees per tile.

The U-Net model recall and F1-scores improved significantly as the number of trees per tile increased (Figure 5). The recall showed a sharp rise from 0.0000 to 0.8623 when the number of trees per tile increased from 1 to 130; the recall continued to improve more gradually beyond this inflection point.

The F1-score also increased rapidly as the number of trees per tile approached 130 and remained relatively stable thereafter (0.81–0.82). The pattern was statistically significant (ANOVA, *p* < 0.001) and these trends, taken as a whole, show how the model was better able to detect tree canopy as more training examples became available.

Precision started high, even with extremely small datasets (i.e., 10 trees per tile), and varied from 0.74 to 0.84 across the remainder of the experiments. The high precision generated in the smallest dataset occurred because the model made very few positive predictions. In this case, the FPs were minimized, and the small number of predictions were mostly correct. As a result, the precision value was inflated. This outcome does not reflect strong model performance but rather indicates a conservative prediction pattern in the early training stages.

Taken together, these results confirm that a sufficiently large and well-distributed training dataset is critical for achieving high-quality tree segmentation and tree detection. Our experiments provide an empirical benchmark indicating that a minimum density of approximately 130 trees per tile corresponds to roughly 16,900 manually annotated tree crowns used for model training and validation, and that this approach is required to achieve stable and reliable model performance. This empirically derived threshold offers a concrete reference point for practitioners planning annotation campaigns with limited time and/or resource constraints.

## 4. Discussion

This study aimed to produce a practical, open, and transferable deep learning framework for mapping urban tree canopy using NAIP imagery. The discussion below starts out by providing some additional model development insights for practitioners, before tackling model performance in City Terrace and what those results would likely mean for model transferability, and closing with a discussion of limitations, future research, and how the framework we have built advances urban forestry best practice.

### 4.1. Model Development Insights for Practitioners

This study provided important lessons on deep learning model development for practitioners. The checkerboard sample design used for training data collection provided a straightforward and replicable method to ensure spatially stratified random sampling across the study area. The analysis of varying training data sizes showed a dynamic trade-off between precision and recall as sample size increased. Small sample sizes produced high precision at the cost of low recall, meaning the rate of TPs in model prediction was high, but the model missed many trees. We therefore increased the sample size to improve the recall, even though precision did not improve significantly. Our analysis showed that using a total of 20,000 annotations (72.7% of all labeled trees) from the 70 gray-shaded tiles in Boyle Heights (Figure 3) was sufficient to train a model that achieved good precision and recall, without significant performance loss compared to using the full dataset.

From a practical perspective, the data sufficiency analysis suggests that, in the context of NAIP-based tree canopy segmentation, annotating approximately 130 trees per 320 × 320 px tile—corresponding to about 16,900 manually annotated tree crowns used for training and validation—is sufficient to reach a plateau in Dice performance around 0.82. These results indicate that reliable canopy maps can be generated without exhaustively labeling all trees, provided annotations are spatially well distributed across the study area. These results also offer important insights for practitioners with specific needs. For example, a model with relatively little sample data may suffice if a high rate of TPs is the priority, whereas a model with larger sample data will be required if the goal is to produce a complete urban tree inventory; of course, the first option will likely incur less effort and cost than the second option.

Lidar-informed models have recently achieved impressive performance in urban forest applications, including sub-meter canopy height mapping over entire states using NAIP imagery as the optical input [64]. While these approaches represent a performance ceiling when lidar is available, they require extensive airborne lidar acquisitions and substantial processing, which remain costly and are still missing for many smaller municipalities in the United States. Our framework is instead designed around free NAIP imagery and open-source tools, providing a practical alternative for cities and towns that lack access to lidar or commercial satellite data.

The large number and variety of deep learning architectures and choices of remote sensing data in the existing literature on mapping urban tree canopy report average accuracies between 70 and 90%. Direct performance comparisons with lidar-assisted or sub-meter-resolution frameworks should be interpreted cautiously, as these studies differ substantially in data source, spatial resolution, urban context, and evaluation protocol. Taking two recent papers as examples, Li et al. [39] used 20 cm resolution imagery and lidar data to achieve a 0.76 F1-score using DeepLapV3+ and SAM model, and Pedley and Morgenroth [65] used 7.5 cm resolution imagery and lidar with a U-Net model and produced a 0.87 F1-score. Our model achieved an F1-score of 0.824 with 60 cm resolution NAIP imagery in place of lidar and this result points to the feasibility of our model.

The study also identified optimal hyperparameters for models using NAIP imagery, including the use of 320 × 320 px tiles, a 50% overlap during training, and comprehensive data augmentation for the U-Net segmentation model. The training process used the Adam optimizer, a combined Binary Cross-Entropy and Dice Loss, a batch size of 4, 50 epochs, and a pixel probability threshold of 0.25. For the YOLOv9e object detection model, the best performance was achieved with an IoU threshold of 0.2 and a confidence threshold of 0.1. The data augmentation involved flipping, rotation, color jittering, contrast adjustment, and normalization. These configurations will help practitioners to reproduce the kinds of results achieved here in their study areas.

### 4.2. Model Performance in City Terrace

We tested the two optimized models in City Terrace to evaluate their generalization performance across different urban areas. The optimized U-Net model achieved a precision of 0.789 and recall of 0.862 (Table 3, row 3), while the YOLO model showed lower performance with a precision of 0.747 and recall of 0.636 (Table 4, row 4). The comparison of model predictions and manual annotations in City Terrace highlights the practical value of our NAIP-optimized workflow. For canopy segmentation, the manually annotated canopy area totaled 561,125 m^2^. The U-Net model correctly identified 453,501 m^2^ (TPs) and 119,680 m^2^ of trees (FPs) at the pixel level. These results show our model predicted 19.2% more tree canopy than the manual annotations. For individual tree detection, the manual dataset includes 19,413 annotated tree crowns, whereas the YOLO model predicted 15,637 bounding boxes. Interpretation of these instance-level results requires caution, as there is not a one-to-one correspondence between bounding boxes and annotated polygons. In practice, multiple bounding boxes may correspond to a single annotated tree, or a single prediction may span multiple crowns. The YOLO predictions showed that 14,786 bounding boxes have centers located within manually annotated polygons, and that 17,197 annotated tree crowns intersected with at least one predicted bounding box.

**Figure 6.**
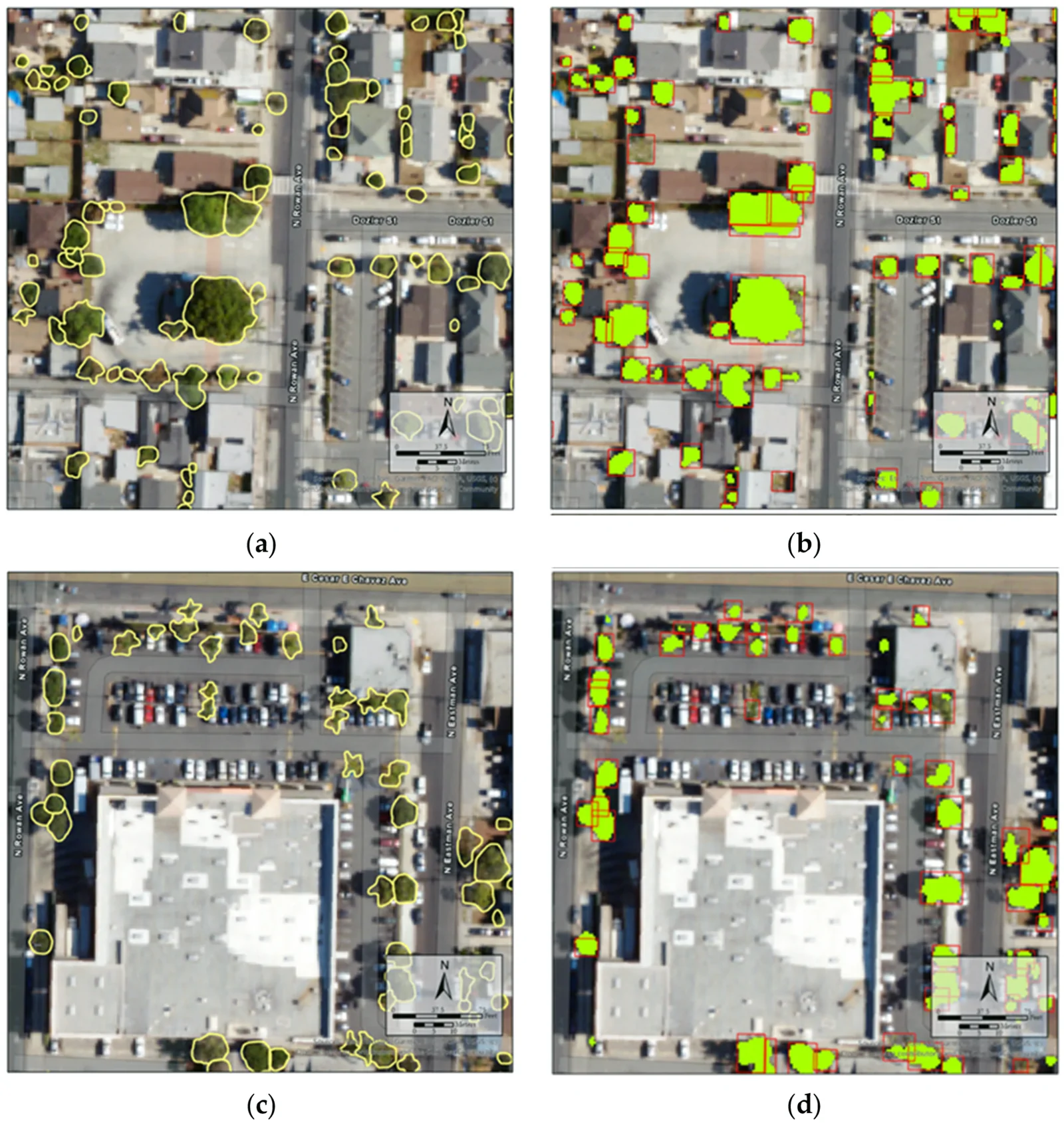
Visual comparison of tree crown detection results in residential (**a**,**b**) and commercial (**c**,**d**) areas. The left column (**a**,**c**) shows manually annotated tree crowns in yellow, and the right column (**b**,**d**) presents predictions from U-Net (segmentation, green) and YOLO (detection, red).

These results show the spatial variability of model performance and the realistic potential and limits of using NAIP imagery and deep learning models for mapping urban tree canopy (Figure 6). Both models (Figure 6c,d) show strong performance in detecting large and well-separated trees, particularly those located in open spaces such as front yards, sidewalks, and parking lots. U-Net effectively segments the full extent of irregular and overlapping crowns, preserving their detailed shapes, and YOLO also performs well in identifying compact and clearly bounded canopies in these conditions. In the residential area (Figure 6a,b), U-Net delineated complex tree crowns more consistently, including those that are irregularly shaped or overlapping. In contrast, YOLO occasionally mis-identified low vegetation, such as identifying grass patches in the upper-left corner of Figure 6a as tree crowns. This type of confusion is rarely observed in the U-Net output. In the commercial area (Figure 6c,d), where trees are often smaller and surrounded by buildings or pavement, neither model detected a visible tree in the central-upper portion of the image. While U-Net captures the overall shape and spatial extent of tree crowns, YOLO complements it by separating closely clustered trees into distinct instances. This instance-level separation is useful for counting individual trees and conducting fine-scale analysis, and the two approaches used together support more detailed and robust tree-level analysis. The complex structure of urban environments remains a challenge, and whilst the 60 cm to 1 m resolution of NAIP imagery is sufficient for capturing large canopies, accurately delineating small trees and low vegetation in dense and heterogeneous landscapes is still difficult.

### 4.3. Model Transferability

Transfer learning has been used to generalize locally trained models and expedite knowledge discovery with remotely sensed imagery [53]. There are numerous challenges related to the spectral variability of features and the cost of acquiring large datasets for model training. One of the primary goals of this study was to evaluate how well a deep learning model leveraging widely accessible, large-scale open-source data such as NAIP imagery in a specific area could be used in other urban environments without requiring additional retraining. This notion of transferability is a critical measure of a model’s practicality and scalability, especially for enabling low-cost, high-quality urban forest mapping in cities with limited data and budgets.

To test this, we designed a transferability experiment by applying the framework trained solely in Los Angeles, CA to two completely unseen cities—San Francisco, CA and Phoenix, AZ—using 2022 and 2021 NAIP imagery, respectively. The San Francisco, CA study area offered a dense, grid-based urban structure characterized by narrow streets, continuous rows of street trees, and strong shadowing effects from buildings, whereas the Phoenix, AZ study area featured larger parcels with sparse vegetation reflecting the dry desert climate.

**Figure 7.**
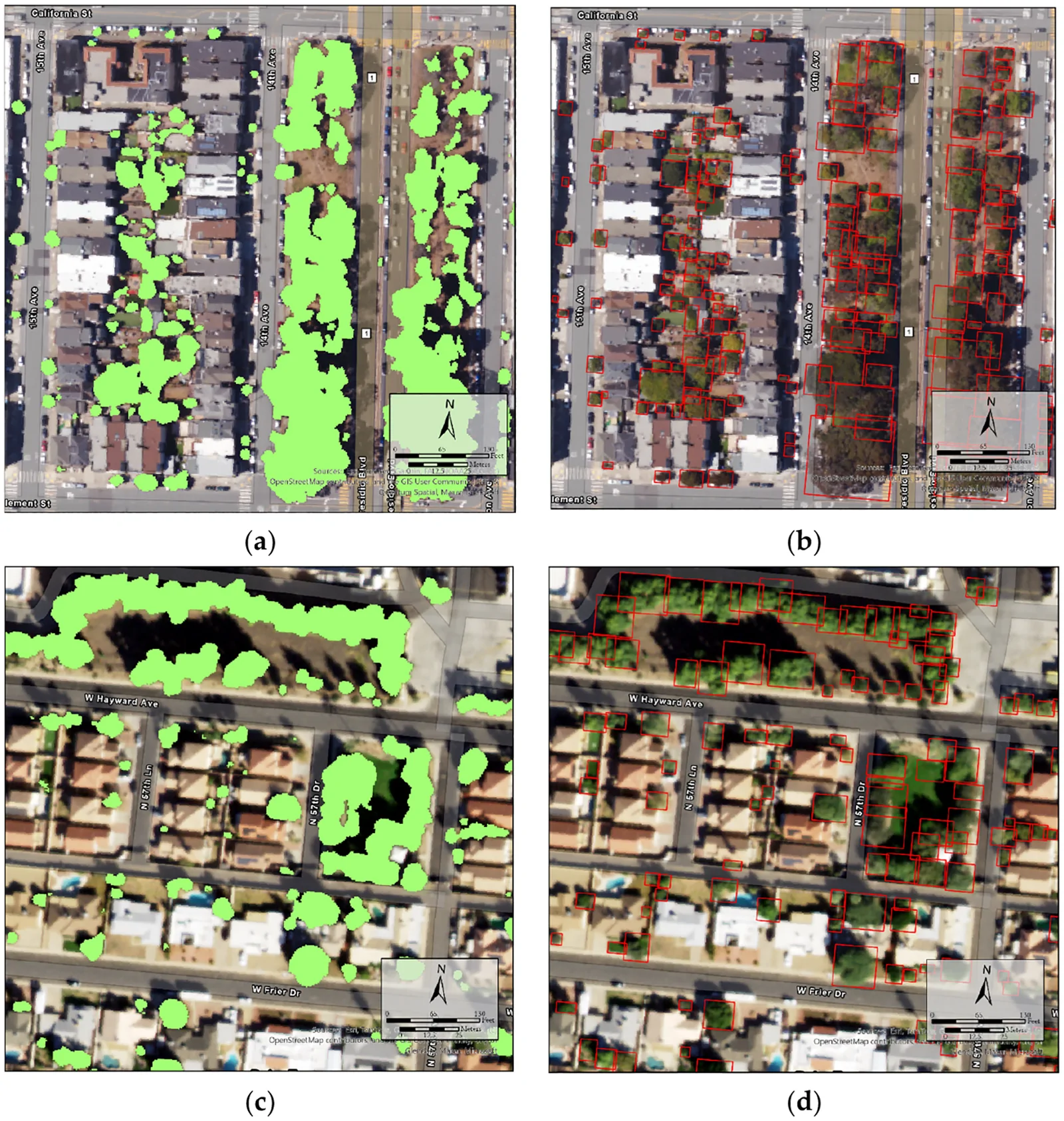
Tree canopy segmentation and object detection in San Francisco, CA (**a**,**b**) and Phoenix, AZ (**c**,**d**). The left column (**a**,**c**) shows pixel-based segmentation results from the U-NET model highlighting tree canopy areas in green and the right column (**b**,**d**) presents instance-level tree detections from the YOLO model using red bounding boxes.

As shown in Figure 7, the framework showed promising transferability in both cities based on visual interpretation. The U-Net model delineated many of the tree canopy shapes in San Francisco’s compact streetscapes and Phoenix’s dispersed residential blocks. It distinguished canopy boundaries even in areas with shadows and over various backgrounds such as asphalt, soil, and grass, while rarely misclassifying low vegetation or shrubs as trees. This indicates that the model learned morphological and spatial features unique to tree crowns, beyond color and texture cues alone. In some densely forested zones, tree boundaries appeared slightly fragmented, but overall segmentation quality remained stable. The YOLO model detected individual trees or clusters within the canopy regions defined by U-Net, even under complex visual conditions where multiple canopies overlapped. The combined strategy of 50% overlap and data augmentation produced a more balanced precision–recall profile. While quantitative differences on the Boyle Heights validation set were modest (Dice range: 0.802–0.824), qualitative visible inspection of San Francisco imagery suggested more improvements in high-density canopy zones, particularly along continuous rows of street trees (Figure 4). The combined strategy is therefore recommended for its improved generalization behavior in geographically diverse urban contexts, even where primary evaluation metrics show limited gains.

These results provide clear practical value for urban forestry practitioners by demonstrating how a NAIP-optimized workflow can support multiple inventory needs within a single framework. The U-Net and YOLOv9e models offer complementary outputs, with segmentation capturing canopy extent and object detection identifying individual trees; taken together, they offer a more robust representation of urban forest structure. The incorporation of data augmentation and 50% tile overlap contributed to improved robustness by mitigating the effects of illumination variability and tile-boundary artifacts commonly present in NAIP imagery.

In addition, the workflow shows encouraging evidence of transferability across urban environments within the southwestern United States. A single model trained in Los Angeles produced consistent and interpretable results when applied to San Francisco, CA and Phoenix, AZ, suggesting that NAIP-optimized configurations can generalize across cities with similar ecological and urban characteristics. While further quantitative validation is needed, these findings indicate that the workflow can reduce the need for extensive retraining and data collection, thereby lowering the cost and effort required to generate urban tree canopy maps in new locations.

By combining deep learning with NAIP imagery, the workflow provided here offers a scalable, cost-effective, and science-based method for supporting urban forest management across multiple scales.

### 4.4. Implications for Urban Forestry Practice

The findings from this research have significant practical implications for urban forestry practice. Traditional tree inventories are labor-intensive, costly, and time-consuming. Our workflow offers a path to automate the creation and updating of city-wide tree inventories with high temporal frequency. This allows municipalities to monitor urban canopy cover goals, assess tree equity across neighborhoods, and manage green infrastructure more efficiently. The two models provide richer and more valuable data for practical decision-making than simple point locations with outdated tree attributes in traditional inventories. The U-Net’s segmentation masks yield precise canopy area and geometry, which are critical inputs for calculating ecosystem services such as stormwater interception, species diversity, carbon sequestration, and heat mitigation. The YOLO object detection model complements the U-Net outcomes by providing discrete tree count estimates, which are often needed to set priorities and budgets for tree planting and maintenance from one year to the next. These estimates need to be interpreted carefully given our results show the YOLOv9e model achieved a best F1-score of 0.687 with a recall of 0.636 on the City Terrace test set (Table 3, IoU = 0.2, confidence = 0.1). These results suggest we should treat YOLOv9e detection outputs as approximate estimates rather than complete enumerations. Future work could explore using the U-Net canopy mask as a spatial prior to constrain the YOLO counts and thereby improve recall in densely vegetated areas.

By providing a detailed and reproducible methodology with open data, this study offers a framework that can be adopted by other municipalities. The ability to apply a pre-trained model to new locations with reasonable success reduces the barrier to entry for cities lacking machine learning expertise. Our approach promotes the standardization of urban forest monitoring, allowing for more meaningful comparisons of canopy health and policy effectiveness across different neighborhoods, cities, and regions.

### 4.5. Limitations and Future Research

While this study presents a structured, reproducible, and NAIP-optimized workflow for estimating urban tree canopy extent and approximate tree counts, several limitations should be acknowledged. First, the spatial resolution of NAIP imagery (60 cm) constrains the detection of small trees; crowns with diameters less than approximately 1.5 m were frequently missed. In addition, dense urban structures, occlusion, and shadowing effects reduce segmentation accuracy in complex environments. Improving the detection of small or partially visible trees remains an important direction for future research. Second, certain applications, such as species classification and tree health assessments, require precise individual crown delineation and additional spectral or structural information that cannot be reliably derived from NAIP imagery alone. Integrating higher-resolution or multi-modal data sources (e.g., lidar or hyperspectral imagery) could help address these limitations. Third, the hyperparameter tuning strategy employed in this study was based on a structured grid search over a set of operationally relevant configurations, rather than more exhaustive optimization approaches such as Bayesian optimization or repeated cross-validation. While this design supports reproducibility and practical implementation, future work could explore more systematic optimization strategies to further improve model performance. Finally, although the workflow demonstrated encouraging qualitative transferability to previously unseen cities within a similar southwestern U.S. ecotone, this assessment was based primarily on visual inspection. A more rigorous, quantitative evaluation across diverse geographic regions is needed to fully characterize model generalization and domain shift [66]. Future research will focus on expanding transferability testing, refining cross-city adaptation strategies, and incorporating emerging approaches such as geospatial foundation models and vision–language models to further enhance automation and scalability in urban tree canopy mapping.

## 5. Conclusions

This paper presents a structured, reproducible, and NAIP-optimized deep learning workflow for urban tree canopy mapping that integrates U-Net segmentation and YOLOv9e object detection to estimate canopy extent and identify trees. Using freely available NAIP imagery and open-source models, the workflow provides a scalable and cost-effective alternative to approaches that rely on lidar or commercial imagery, while achieving competitive performance for urban tree mapping applications.

The results demonstrate that the optimized U-Net model achieved a Dice coefficient of 0.824 for canopy segmentation, while the YOLOv9e model achieved an F1-score of 0.687 for individual tree detection. Model performance was strongly influenced by NAIP-specific design choices, with optimal results obtained using 320 × 320 px tiles, 50% overlap, and data augmentation. In addition, a data sufficiency analysis showed that performance stabilizes when approximately 130 trees are annotated per tile, providing a practical benchmark for training data collection. Together, these findings offer empirically grounded guidance for reproducible model development using NAIP imagery.

The workflow also shows encouraging evidence of transferability, as models trained in Los Angeles produced consistent results in previously unseen urban environments without retraining. Although further quantitative validation across diverse geographic regions is needed, these results suggest that NAIP-optimized workflows can be extended to new cities with minimal additional effort.

Overall, this study advances a structured, bottom-up framework that bridges the gap between complex deep learning methods and the operational needs of urban forestry practitioners and managers. By emphasizing accessibility, reproducibility, and NAIP-specific optimization, the proposed workflow enables practitioners to generate timely, consistent, and scalable urban tree canopy products, supporting data-driven decision-making for the sustainable management of urban green infrastructure.

## Figures and Tables

**Table 1.** U-NET and YOLO model performance across various tile sizes.

Model	Tile Size (px)	Precision	Recall	Dice/F1-Score
U-Net	80 × 80	0.790	0.862	0.824
	160 × 160	0.788	0.861	0.823
	320 × 320	0.789	0.862	0.824
	640 × 640	0.810	0.820	0.815
YOLO	80 × 80	0.679	0.686	0.681
	160 × 160	0.734	0.621	0.672
	320 × 320	0.747	0.636	0.687
	640 × 640	0.734	0.630	0.678

**Table 2.** U-NET performance across various pixel-probability thresholds (τ).

Threshold	Precision	Recall	F1-Score	Threshold	Precision	Recall	F1-Score
0.05	0.709	0.921	0.801	0.25	0.789	0.862	0.824
0.10	0.743	0.899	0.814	0.50	0.828	0.816	0.822
0.15	0.763	0.885	0.819	0.85	0.883	0.719	0.793
0.20	0.777	0.873	0.822	0.90	0.896	0.686	0.777

**Table 3.** Performance of YOLOv9e model across various IoU and confidence thresholds on the City Terrace test dataset (100 files and 17,466 annotated trees).

Model	IoU Threshold	Confidence Threshold	Precision	Recall	F1-Score
YOLO	0.1	0.1	0.679	0.686	0.681
	0.1	0.2	0.760	0.501	0.604
	0.2	0.1	0.747	0.636	0.687
	0.2	0.2	0.755	0.629	0.685

**Table 4.** Performance of four U-NET models under various combinations of overlap and augmentation (Augme.) using TPs, TNs, FPs, FNs, precision, recall, and the Dice coefficient.

Overlap (50%)	Augme.	TPs	TNs	FPs	FNs	Precision	Recall	Dice
No	No	1,119,350	8,641,294	300,198	179,158	0.789	0.862	0.824
Yes	No	1,103,471	8,656,138	285,354	195,037	0.795	0.850	0.821
No	Yes	1,177,782	8,480,368	461,124	120,726	0.719	0.907	0.802
Yes	Yes	1,097,845	8,668,977	272,515	200,663	0.801	0.845	0.823

## Data Availability

The original data and Python source code used in this study are openly available on Zenodo at https://doi.org/10.5281/zenodo.17459767 and Github at https://github.com/uscssi/urban-tree-canopy, respectively. These materials are also shared as an ArcGIS deep learning model, entitled “Urban Tree Segmentation with NAIP Imagery,” at https://www.arcgis.com/home/item.html?id=a44398820d1a4fbaa6bfeedb8d1559a5.

## Highlights

### What are the main findings?

- The open-source U-Net segmentation and YOLOv9e object detection models were optimized using National Agriculture Imagery Program (NAIP) imagery to map urban tree canopy.
- A series of experiments demonstrated that the workflow is structured and reproducible, and showed encouraging evidence of transferability to previously unseen urban areas without retraining.

### What is the implication of the main finding?

- The resulting NAIP-based accuracies fall within the range reported for lidar- and commercial imagery-based urban tree canopy studies, while relying solely on freely available aerial imagery and open-source models.
- By reducing data cost and technical barriers, the approach enables scalable and accessible urban tree canopy mapping for planners, practitioners, and researchers.

## Abbreviations

The following abbreviations are used in this manuscript:

ANOVA: One-way Analysis of Variance
C2f: Cross-stage Partial-fusion
CIR: Color-infrared aerial photography
CNN: Convolutional Neural Network
FN: False Negative
FP: False Positive
GPU: Graphic Processing Unit
IoU: Intersection over Union threshold
NAIP: National Agriculture Imagery Program
NIEHS: National Institute of Environmental Health Sciences
NIR: Near Infrared Light
NLCD: National Land Cover Database
PGI: Programmable Gradient Information
R-CNN: Region-based Convolutional Neural Network
RGB: Red, Green, and Blue
TP: True Positive
U-Net: Fully Convolutional Neural Network
USDA: U.S. Department of Agriculture
YOLO: You Only Look Once
